# Searching for a HIV-1 Cure

**DOI:** 10.7150/thno.124358

**Published:** 2026-01-01

**Authors:** Chen Zhang, Bharat N. Chaudhary, Mohammad Uzair Ali, Mary G. Heser, Swara S. Patel, Xiaoqing Du, Soumya S. Dey, R. Lee Mosley, Sudipta Panja, Howard E. Gendelman

**Affiliations:** Department of Pharmacology and Experimental Neuroscience, University of Nebraska Medical Center, Omaha, NE, USA 68198-5880.

**Keywords:** CRISPR, CRISPR-Cas9, CCR5, CXCR4, CD4, Gene Delivery, HIV-1 suppression, Viral resistance, Combination antiretroviral therapy, HIV-1 replication

## Abstract

Advances in stem cell transplantation, broadly neutralizing antibodies, ultra-long-acting antiretroviral drugs, therapeutic vaccines, cell engineering, gene therapy, and immune-based molecular therapies have highlighted efforts to cure human immunodeficiency virus type 1 (HIV-1). However, none of these are durable. The challenges to achieve complete viral eradication include durability, accessibility, delivery, off-target effects, and practicality. All approaches, including combinatorial therapies with ultra-long-acting antiretrovirals, broadly neutralizing antibodies, or latency-reversing agents, which reduce reservoir size and achieve durable viral suppression, are discussed. These include key translational insights for clinical relevance. Among these, gene-editing technologies have shown promise in disrupting proviral DNA and delivering targeted therapies by targeting latent reservoirs and host viral receptors. This review examines the scientific, clinical, and ethical considerations, focusing on direct viral excision and co-receptor editing as key strategies for a viral cure.

## 1. Introduction

### 1.1 Insights of historical relevance from HIV-1 discovery to the present day

Human immunodeficiency virus type 1 (HIV-1) infection causes acquired immunodeficiency syndrome (AIDS). AIDS was first recognized in the early 1980s as a life-threatening condition that primarily affects men who have sex with other men 1. The syndrome is characterized by the rapid onset of opportunistic infections, such as *Pneumocystis* pneumonia, and rare cancers, such as Kaposi's sarcoma. In 1983, scientists at the Pasteur Institute, led by Drs. Luc Montagnier and Françoise Barré-Sinoussi isolated a retrovirus from the lymph node biopsies of an individual with AIDS 2. This virus was first identified as the lymphadenopathy-associated virus (LAV) and human T-lymphotropic virus type III (HTLV-III), both of which selectively target CD4^+^ T cells. In 1984, Dr. Robert Gallo and colleagues at the US National Institutes of Health (NIH) confirmed that the isolated virus was responsible for AIDS 3. Subsequent studies elucidated the virus structure, function, and genetic composition and renamed it HIV-1. This discovery marked a significant milestone in establishing the cause of the disease, facilitating successful diagnosis, and ultimately leading to effective antiretroviral (ARV) drug treatment 4. From 1984 to 1986, recombinant viral clones were ultimately created, leading to the creation of full-length molecular clones of HIV-1 5-7. Molecular and biochemical analyses have confirmed that HIV-1 has nine key genes in the viral RNA gene sequence that are associated with the regulation of replication, control, and structural modulation during its life cycle 8, 9.

The principal structural genes of HIV-1 are *gag*, *pol*, and *env*. The *gag* gene encodes the core p24 (viral capsid) and p17 (matrix) proteins. The *pol* gene encodes viral enzymes, all of which are essential for completing the viral replication cycle, including reverse transcriptase, viral integrase, and protease. The *env* gene encodes the gp160 envelope glycoprotein, which is cleaved into gp120 and gp41, two proteins crucial for viral attachment, fusion, and entry into host cells 10, 11. The viral genome also includes accessory genes, such as *tat, rev, nef, vif, vpr*, and *vpu*, in addition to the primary structural genes. These genes encode proteins that regulate transcription, facilitate immune evasion, and enhance viral replication 11, 12.

**Figure 1:**
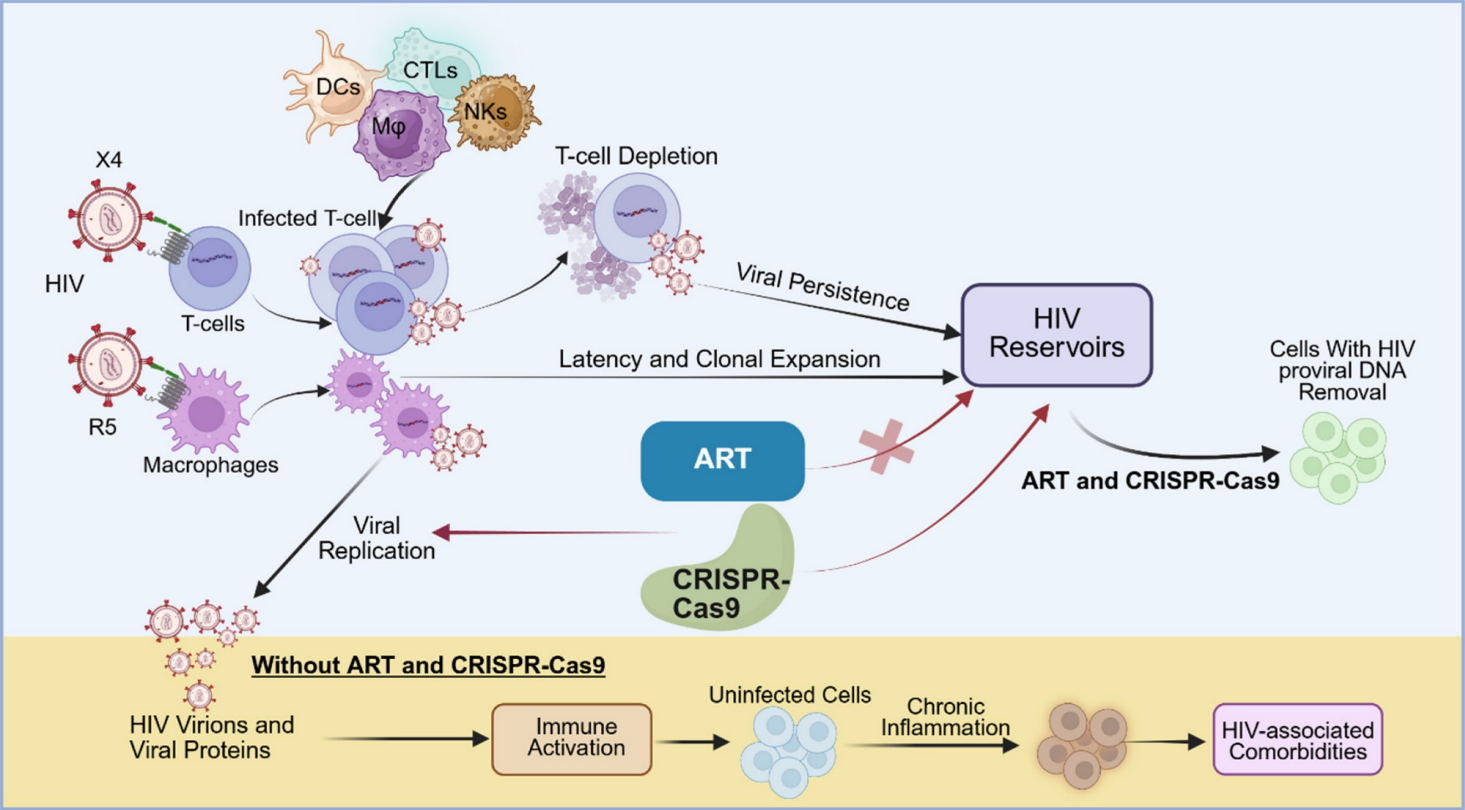
** Current ART and CRISPR-Cas9 treatment strategies.** Tissue and cell reservoirs of HIV-1 infection (top): CXCR4 (X4)- and CCR5 (R5)-tropic HIV-1 strains preferentially infect CD4^+^ T cells and macrophages, respectively, and can persist in a latent state within the genome of myeloid and T cells. These cellular reservoirs undergo productive replication or remain transcriptionally silent under active immune surveillance and potent antiretroviral therapy (ART). Homeostatic proliferation of latently infected immune cells in the absence of conventional activation signals leads to their clonal expansion. Upon activation, these latent cells can reactivate and produce new virions; both processes contribute to the maintenance of viral persistence in the host. Viral persistence and immune activation (bottom): Chronic immune activation and inflammation occur in both infected and bystander immune cells, which promote progressive destruction of cells and tissues. Although viral replication is effectively suppressed during ART, treatment interruption results in rapid viral reactivation and rebound, which further causes severe immune and tissue damage. CRISPR-Cas9-based therapeutic strategies aim to target and excise integrated proviral DNA from reservoir cells, thereby preventing viral rebound even after ART cessation. Created with BioRender.com.

**Figure 2:**
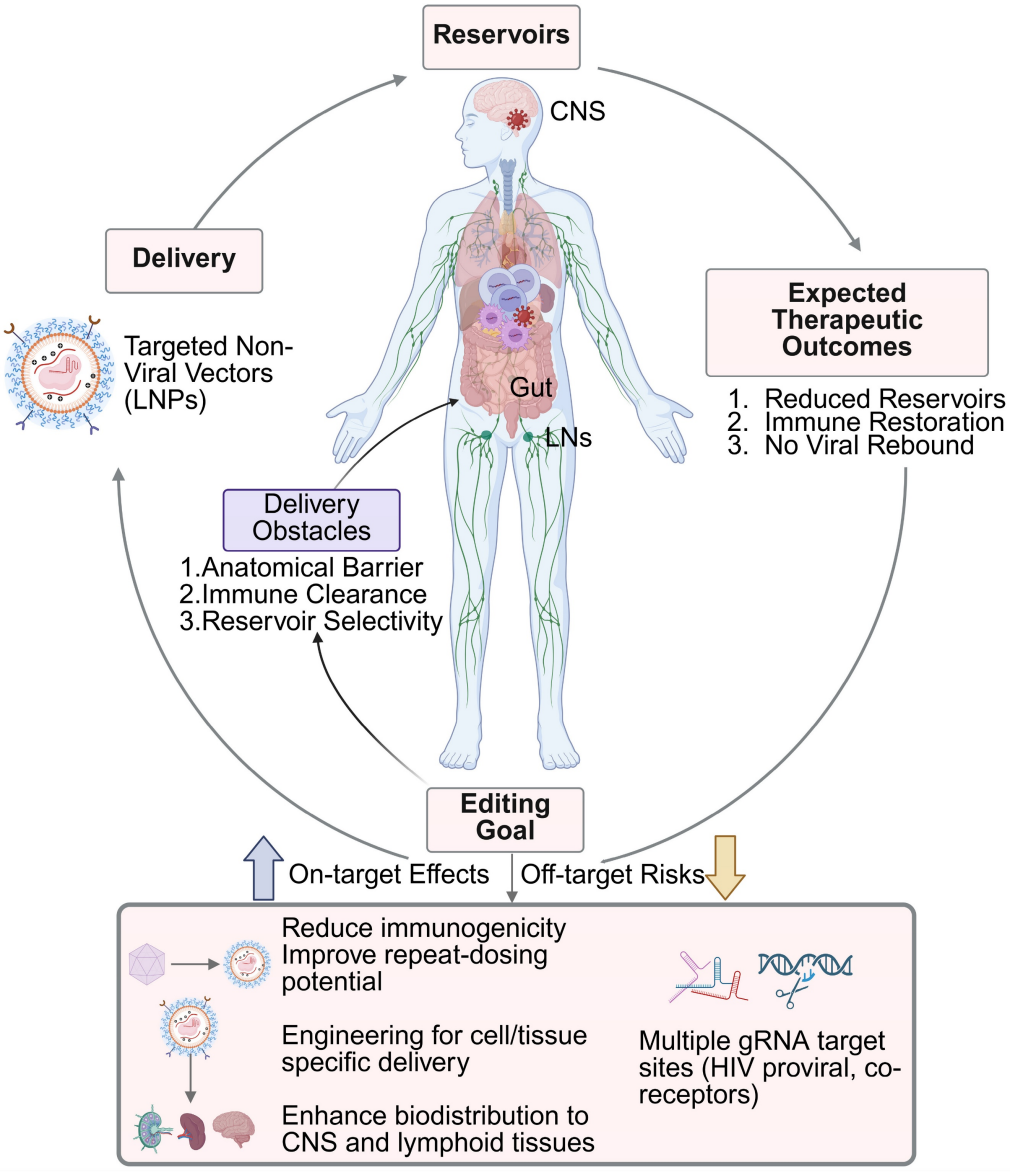
** Gene delivery and HIV-1 reservoir elimination strategies.** HIV-1 reservoirs are protected by anatomical and immunological barriers, which hinder the efficient delivery of gene-editing tools. Local delivery obstacles include poor biodistribution, immune clearance, and off-target accumulation. Current delivery system under development aims to: (1) reduce immunogenicity and improve repeat-dosing; (2) enable cell- and tissue-specific targeting; and (3) enhance biodistribution to the CNS and lymphoid tissues. Additionally, multiple gRNA target sites are needed to excise integrated HIV proviral DNA as well as co-receptor genes such as CCR5 or CXCR4. Together, these advancements aim to enhance *in vivo* editing efficiency and achieve long-term viral remission or a functional cure. Created with BioRender.com.

The viral life cycle begins with the binding of the viral glycoprotein gp120 to the primary receptor CD4, followed by attachment to the co-receptors C-C chemokine receptor 5 (CCR5) or C-X-C chemokine receptor type 4 (CXCR4), which are found on the surface of host CD4^+^ T and myeloid cells 13. After receptor engagement, a conformational change is triggered by the gp120-CD4 complex, which exposes the binding site of the envelope glycoprotein gp41, mediating the insertion of the viral envelope into the host cell plasma membrane 14. Once inside the cell, the viral proteins are released into the cytoplasm, and reverse transcriptase reverse transcribes the single-stranded (ss) viral RNA into double-stranded (ds) viral DNA 15. This newly formed viral DNA is transported into the nucleus and integrated into the host genome to form replication-competent proviral DNA 16. Subsequently, the host's transcription and translation machinery is used to synthesize and express viral proteins. If the infected cell is activated after therapeutic interruption, newly synthesized viral proteins can assemble into viral particles and be released from the cell as immature virions. These viral particles are then cleaved by HIV-1 protease, which processes viral polyproteins, leading to the formation of mature, infectious virions 17, 18. The biggest obstacle to HIV elimination is the persistence of these latent viral reservoirs. Shortly after infection, HIV integrates its RNA genome into the host DNA, forming proviral DNA primarily within CD4^+^ T cells. These cells can remain dormant or expand through clonal proliferation for years, allowing the virus to evade the immune surveillance and remain unaffected by antiretroviral therapy (ART) (**Figure 1**) 19-22. Additionally, viral reservoirs vary and exist not only in different cell types (mainly CD4^+^ T and myeloid cells) but also across various tissues in the body, such as the lymph nodes (LNs), gut, bone marrow, and central nervous system (CNS) (**Figure 2**) 23-25.

### 1.2 Role of HIV-1 Co-receptors in Pathways for Viral Control

The CD4 receptor is a cell-surface protein mainly found on T lymphocytes and myeloid cells that serves as the primary docking site for HIV-1 13. Anti-CD4 nanobodies, such as Nb457, have demonstrated strong potency against diverse HIV-1 genotypes with no reported cytotoxic effects 26. After HIV binds to the CD4 receptor on T cells, it requires a co-receptor, either CCR5 (for R5-tropic viruses) or CXCR4 (for X4-tropic viruses), or both, to enter the cell (**Figure 1**). These are G protein-coupled receptors (GPCRs) from the chemokine family 27-29. Affinity for specific co-receptors affects cell tropism, viral entry, replication, and disease progression. In terms of structural organization, they share key common features, including an extracellular N-terminal domain, three extracellular loops (ECL1-3), and three intracellular loops 30.

The cellular expression patterns of these HIV-1 coreceptors overlap. CXCR4 is broadly expressed on most immune cells (T cells, B cells, and monocytes) and stem cells (bone marrow stem and progenitor cells) 31-33. CCR5 has a more specialized and selective expression in activated T cells, macrophages, dendritic cells, endothelial cells, vascular smooth muscle cells, mesenchymal stromal cells, and brain-resident microglia 34-36. However, peripheral blood analysis of HIV-1 infected individuals suggests an upregulation of CCR5 and downregulation of CXCR4 on both CD4^+^ and CD8^+^ T cells 37. This observed effect might be due to the immune environment, especially the presence of inflammatory cytokines such as IL-6, IFN-γ, and granulocyte-macrophage colony-stimulating factor (GM-CSF), which can boost CCR5 expression levels, making mature and activated immune cells more susceptible to infection by R5-tropic HIV strains.

**Figure 3:**
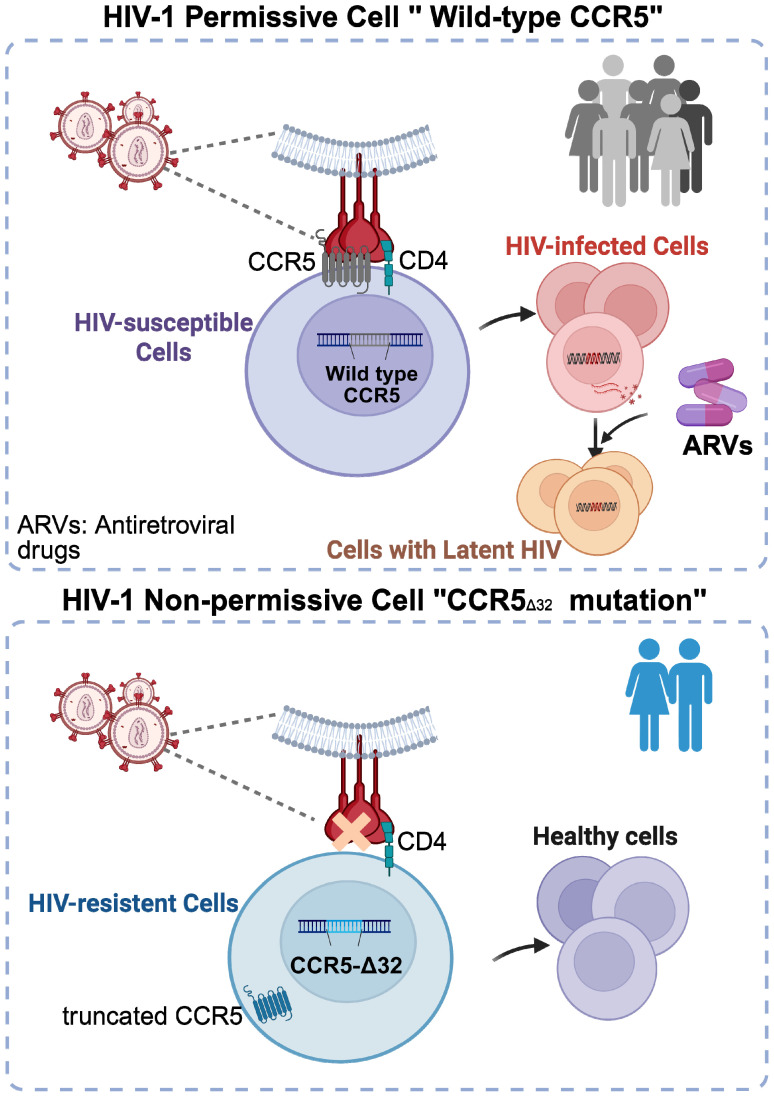
** CCR5-Δ32 mutation confers resistance to HIV-1 infection.** Wild-type CCR5 (top panel): CCR5 is one of two co-receptors required for HIV entry. The HIV envelope protein, gp120, first binds to the CD4 receptor on the surface of susceptible T cells and macrophages. This binding induces a conformational change in gp120, exposing a binding site for CCR5. Subsequent interaction with CCR5 triggers gp41 to unfold and expose its hydrophobic fusion peptide, which inserts into the host membrane. This facilitates fusion of viral and host membranes, allowing viral entry. CCR5-Δ32 mutation (bottom panel): This 32-base-pair deletion leads to a frameshift that produces a non-functional CCR5 protein that is retained in the endoplasmic reticulum, preventing its expression on the cell surface. As a result, R5-tropic HIV strains cannot infect CD4^+^ T cells and myeloid cells. Individuals with homozygous CCR5-Δ32/Δ32 genotypes exhibit near-complete protection from HIV-1 infection, whereas heterozygous individuals show reduced viral susceptibility. The discovery of the CCR5-Δ32 mutation has inspired gene-editing and therapeutic strategies aimed at blocking or mutating the CCR5 receptor to achieve viral elimination. Created with BioRender.com.

Strategies targeting CCR5 and CXCR4 are used for HIV-1 prevention and treatment. These include the design of co-receptor antagonists and gene-based therapies. Technologies such as clustered regularly interspaced short palindromic repeats-associated protein 9 (CRISPR-Cas9) can disrupt CCR5 or CXCR4 genes, rendering cells resistant to HIV by blocking co-receptor binding (**Table 1**) 38. For instance, the application of CRISPR-Cas9 can induce premature stop codons at the CCR5 locus to prevent its expression 39. CCR5 and CXCR4 have immunoregulatory functions that influence host defense and tissue inflammation, which can also affect HIV-1 replication and sustenance of infection 40. Studies of HIV-1 elite controllers suggest that they may carry a 32-base-pair deletion in the CCR5 gene (CCR5-Δ32), a mutation that reduces the surface expression of the CCR5 receptor (**Figure 3**) 29, 41.

Both co-receptors undergo developmental regulation through genetic switches, and HIV exploits these natural patterns by adapting to the most available receptors 42, 43. Understanding the functional dynamics of each HIV-1 co-receptor has enabled the development of effective treatment strategies. For example, maraviroc, which was approved in 2007, is the first CCR5 antagonist that blocks the entry of R5-tropic strains into host cells 44. Using a similar strategy targeting the CXCR4 co-receptor, nanobodies were generated from llamas immunized with cells expressing intact CXCR4. Using a counterselection approach, nanobodies that effectively inhibit HIV-1 entry and chemotaxis *in vitro* have been successfully isolated 45. Using the natural CCR5-Δ32 mutation as a template, current gene therapy approaches have been used for experimental HIV cures, with some people living with HIV (PLWH) achieving long-term viral control after receiving genetically modified immune cells with mutated CCR5 receptor expression 46-49. Although these strategies have shown promising results, they are limited by challenges such as incomplete editing, co-receptor specificity, and potential off-target effects 50. Further mechanistic and therapeutic investigations are required to develop strategies that simultaneously target multiple co-receptors.

The mechanisms aimed at eliminating HIV-1 infection have been previously reviewed 16, 51. Prior studies have largely focused on selectively targeting reservoir cells based on their immunological characteristics. These reports identified vulnerable stages in the viral life cycle that offer therapeutic potential 52. Therefore, this review emphasizes the persistent challenge of residual viremia despite combinatorial ART and various immunomodulating strategies. We further examined emerging preclinical strategies that utilize CRISPR-based systems to excise latent viral DNA using targeted delivery approaches. In addition, this review examines the factors that influence therapeutic outcomes in strategies such as shock-and-kill, viral co-receptor disruption, and viral excision, both individually and in combination. This review highlights key knowledge gaps and emphasizes the need to bridge preclinical advances with clinical translation. Finally, we outline the potential synergistic benefits of integrating these approaches to overcome existing barriers and advance toward a functional HIV-1 cure.

## 2. Pathways for HIV elimination

**Figure 4:**
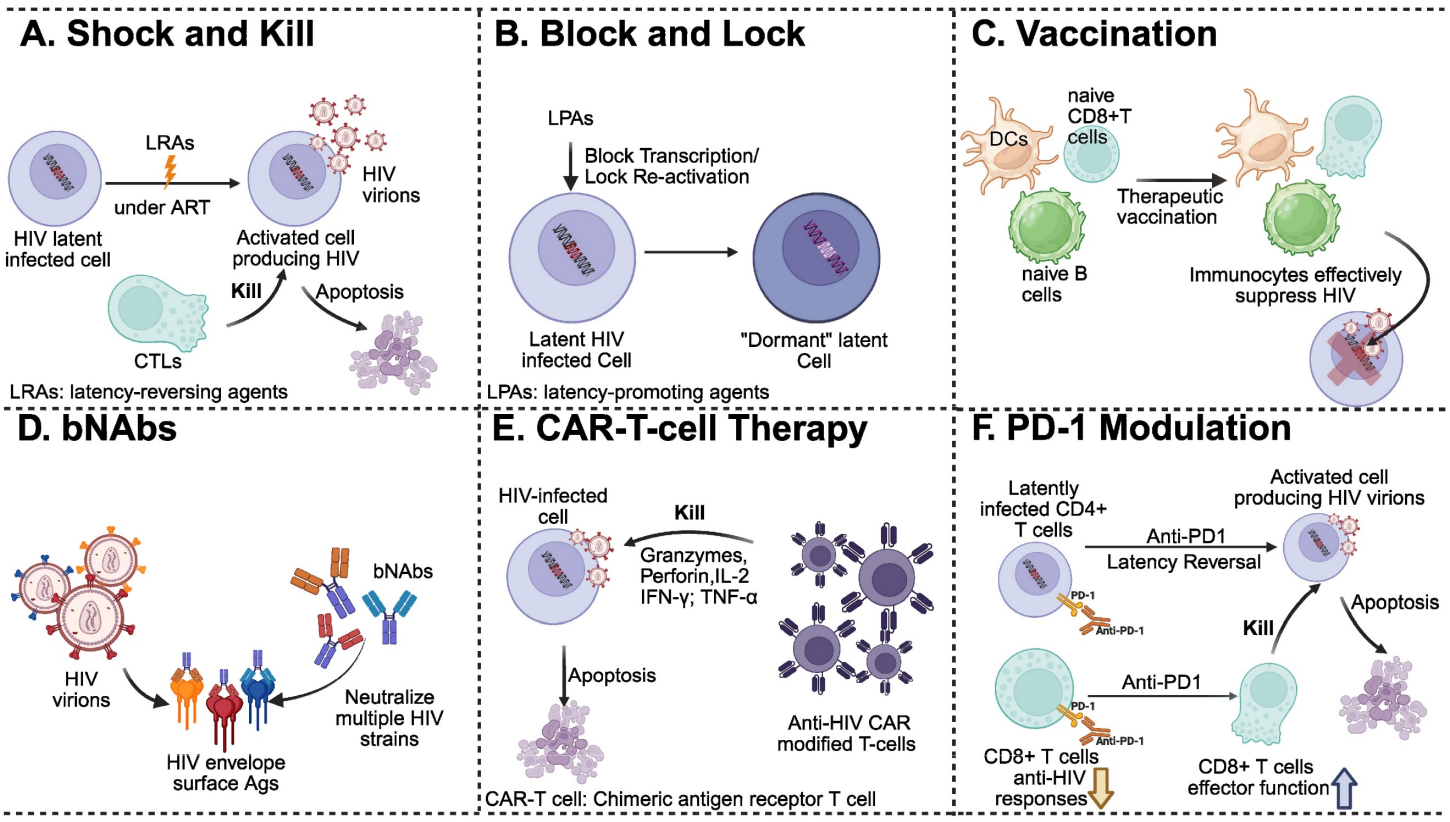
** Current HIV elimination strategies**. **A) The shock-and-kill** strategy targets latent viral reservoirs using LRAs to “shock” the dormant virus into reactivation and render the virus detectable. The active virus then becomes vulnerable to attack by immune effector cells, like cytotoxic T cells (CTLs), or other treatments that “kill” infected cells or induce apoptosis. **B) Block and lock** approaches employ latency-promoting agents (LPAs) to permanently silence or control latency to prevent reactivation and rebound. This method triggers epigenetic changes that “block” viral transcription and “lock” the integrated virus into a dormant state, making reactivation impossible even if treatment is stopped. **C) Vaccination** strategies aim to stimulate or enhance humoral and/or cell-mediated immune responses to regulate viral replication and ultimately eradicate reservoirs. These strategies involve designing HIV immunogens, with or without adjuvants, that activate dendritic cells (DCs) to stimulate naïve CD8^+^ T cells to become CTLs, or B cells to develop into plasma cells that produce HIV-specific antibodies (Abs) capable of killing HIV-infected cells. **D) Broadly neutralizing antibodies (bNAbs)** are a special type of Abs that can neutralize numerous HIV strains. They target conserved regions on the viral surface, such as Env proteins. Unlike naturally induced antibodies, bNAbs are typically engineered and administered passively rather than produced through vaccination. **E) Chimeric antigen receptor (CAR) T-cell therapy** involves genetic constructs that encode antigen-recognition chains, often derived from HIV-specific Abs, used to modify T cells of PLWH. These HIV-specific CAR T cells recognize and eliminate HIV-infected cells, including viral reservoirs, through cytotoxic mechanisms that induce apoptosis. **F) Programmed death-1 (PD-1) modulation** strategies use anti-PD-1 antibodies to target exhausted T cells expressing PD-1, aiming to either remove these T cells or restore their impaired effector functions to boost virus-specific immunity. Created with BioRender.com.

Advancements in ART have transformed HIV infection from a fatal disease into a manageable chronic condition 53, 54. However, a functional cure for HIV remains out of reach, mainly due to the persistence of latent reservoirs 54, 55. In addition, HIV employs multiple immune evasion strategies that further complicate its eradication. One of the biggest challenges is that HIV directly targets CD4^+^ T cells, gradually depleting key components of the immune response 56. Infected cells downregulate major histocompatibility complex class I (MHC-I) expression, hindering recognition by cytotoxic T lymphocytes 57. The virus can also reside in immune-privileged sites, such as the brain, LNs, and gut-associated lymphoid tissue (**Figure 2**) 57. Over time, immune exhaustion develops, and the high mutation rate of HIV enables it to escape previously effective immune responses 58. Therefore, lifelong ART is required in PLWH. However, long-term therapy presents challenges related to adherence, toxicity, cost, and the emergence of drug-resistant viral strains 59, 60. The burden of comorbidities and limited treatment options further underscore the urgent need for curative strategies in PLWH. Two major therapeutic outcomes are generally envisioned: a functional cure and a sterilizing cure. Functional cure or medicine-free remission is defined as the sustained remission of viral replication in the absence of ART, without evidence of viral transmission, whereas a sterilizing cure represents the complete eradication of replication-competent HIV from the body (**Figure 2**) 61-64. Whether the goal is sterilization or functional cure, researchers have sought to achieve these outcomes through diverse approaches (**Figure 4**), including immune-based interventions that enhance host antiviral responses and gene-editing strategies designed to disrupt key viral or host genomic elements required for infection and persistence 65, 66.

### 2.1 Immune-based therapies for HIV-1 elimination

#### 2.1.1 Shock and kill

Although the “shock and kill” strategy is one of the most widely studied methods aimed at eliminating latent HIV reservoirs to achieve a sterilizing cure, it has limitations. Using latency-reversing agents (LRAs) to “shock” the virus out of its dormant state makes the previously hidden HIV visible to the immune system, which then “kills” the infected cells 67. Since this approach was first proposed as a potential HIV-1 cure, researchers have identified more than 160 small-molecule compounds as LRAs 68-70. For example, plasma-derived exosomes from rhesus macaques selectively fuse with TCR-activated T cells and induce HIV reactivation in resting CD4^+^ T cells; however, immune control remains ineffective. This is evidenced by increased viral transcription, despite the presence of residual virus 71. Moreover, while exosomal delivery of HIV-1 Tat showed some efficacy in reversing latency in primary CD4^+^ T cells, such LRAs have had limited success 72. Therefore, to effectively eliminate HIV, a synergistic combination of the “shock and kill” strategy and additional complementary approaches must be employed. One such approach involves CD3-targeted adenoviral vectors carrying dCas9-VPR CRISPR activators to trigger suicide gene activation, leading to latency reversal and subsequent viral elimination. This method achieved a modest 57.7% reduction in cells producing productive infection, along with only a 2.4-fold increase in cell death 73. In addition, *ex vivo* studies have explored the synergistic combination of IL-15 with HODHBt, a small-molecule inhibitor of protein tyrosine phosphatases 74. Enhanced HIV-specific granzyme B-releasing T-cell responses were observed in PBMCs from HIV-infected individuals receiving ART, suggesting potential roles in latency reversal and immune activation 74. Additionally, using a synthetic bryostatin 1 analog and PKC modulator, SUW133, followed by two rounds of natural killer cell treatment during ART in a humanized HIV-infection model, can delay viral rebound after ART interruption 75. Four out of ten dual-treated mice showed no detectable HIV DNA in their tissues. Each approach highlights the potential of combining LRAs with innate immune effectors to improve HIV clearance. Building on this, studies in simian immunodeficiency virus (SIV)-infected ART-suppressed rhesus macaques have shown that combining the latency-reversing agent AZD5582 with SIV-Env-specific RhmAbs more effectively eliminates proviral DNA from lymphoid reservoirs 76. However, while there were significant reductions in SIV-DNA in lymph node CD4^+^ T cells, complete viral elimination was not achieved in this study. Ongoing research involves the use of hybrid polylactic-co-glycolic acid nanoparticles to encapsulate LRAs for HIV-1 reactivation in infected CD4^+^ T cells. Among these, ingenol-3-angelate has shown promise as it can induce sustained CD4^+^ T cell activation with low toxicity 77. Nonetheless, several translational efforts have led to clinical evaluation. For example, a phase 2 clinical trial (NCT05129189) conducted in 2024 involved PLWH on long-term ART 78. This study tested the effectiveness of combining the anti-PD-L1 antibody ASC22 with Chidamide to reverse HIV latency. Significant increases in cell-associated HIV RNA levels were observed; however, the levels of integrated viral DNA remained unchanged. Based on earlier research, the co-administration of broadly neutralizing antibodies (bNAbs) with LRAs was tested to promote HIV-1 clearance. A phase 2a clinical trial analyzed the effect of the antibody 3BNC117 combined with romidepsin, a histone deacetylase inhibitor used for latency reversal 79. A delayed viral rebound was observed during analytical treatment interruption (ATI); however, the overall difference was not statistically significant. Despite these efforts, none of these approaches have resulted in a functional HIV-1 cure. The major limitation of the “shock” stage is that current LRAs fail to reactivate all latent proviruses, leaving a significant fraction of intact, replication-competent proviruses non-inducible even after multiple or serial administrations 67. In the “kill” stage, immune responses are often too weak or exhausted in ART-treated PLWH to effectively clear the reactivated cells 80, 81. Moreover, some LRAs can trigger widespread immune activation or toxicity, reducing their suitability for long-term use 82. Together, the lack of highly potent and safe LRAs limits the reliability of the shock-and-kill strategy as a curative approach (**Figure 4**). Although LRAs alone or in combination with ART are insufficient to achieve a functional cure, integrating potent immune boosters during the “kill” stage or employing gene-editing strategies that target both latent proviruses and newly reactivated viruses may offer promising potential for a functional cure in PLWH 64, 83.

#### 2.1.2 Block and lock

The “block and lock” strategy deploys a means to permanently silence the virus rather than reactivating it 84. Instead of flushing HIV out of latency, this method uses drugs or genetic tools to “block” the virus in a deeply dormant state, preventing reactivation and replication, even in the absence of ART 84, 85. Exosomes, immune-tolerant and highly penetrant nanoscale carriers, have been​ utilized to deliver RNA cargo for epigenetic silencing of HIV-1 in infected cells 86. When combined with conventional ART, this strategy may reduce dependence on strict treatment adherence in PLWH. This functional cure strategy targets the transcriptional and epigenetic regulatory machinery of HIV-1. Notably, two major categories of block-and-lock agents have been developed: those that disrupt key transcriptional regulators (e.g., Tat inhibitors such as didehydro-cortistatin A (dCA)) and those that modulate epigenetic silencing mechanisms at the HIV promoter level (e.g., RNA-induced silencing and lens epithelium-derived growth factor p75 (LEDGF/p75)-IN interaction inhibitors (LEDGINs)) 87-92. However, the challenge lies in the complete and consistent silencing of all proviral DNA across various tissues and reservoir sites. Many current compounds do not fully penetrate all cellular reservoirs or maintain the long-term suppression of viral replication necessary for complete silencing; thus, the block-and-lock strategy does not permanently silence HIV 93.

#### 2.1.3 Therapeutic vaccines

Therapeutic vaccines have been developed to stimulate the immune system in PLWH, aiming to improve their ability to recognize, control, or even eliminate the virus in combination with ART 94. Unlike preventive vaccines, these vaccines are designed to strengthen the body's natural defenses, suppress viral levels, and achieve viral clearance 94. A major challenge is that HIV rapidly mutates and evades the immune system of the host. Additionally, immune exhaustion caused by chronic HIV infection reduces vaccine efficacy 95, 96. Latent reservoirs are also unaffected by these immune responses, allowing the virus to persist 23. Recently, first-in-human studies of the HIVconsvX T-cell vaccine demonstrated its safety and broad immunogenicity in HIV-negative adult patients. In this phase 1 trial, all participants produced strong T-cell responses that recognized multiple conserved HIV-1 epitopes across the major global clades. Vaccine-induced T cells showed functional versatility, proliferated upon antigen re-exposure, and inhibited HIV-1 *in vitro*, supporting the potential of T-cell-focused immunogens as a therapeutic strategy to target HIV reservoirs and improve viral control 97. To date, therapeutic vaccines have not resulted in durable viral control 98.

#### 2.1.4 Broadly neutralizing antibodies

Broadly neutralizing antibodies (bNAbs) are designed to target the hard-to-reach regions of the HIV envelope, allowing the neutralization of several viral strains 99. They have been used in both preventive 100-102 and therapeutic settings 103, 104, often alongside ART to reduce viral load and delay viral rebound. Preclinical studies have demonstrated that combining bNAbs with ART or LRAs significantly delays viral rebound in humanized mice 105 and macaques 106. Notably, in simian-human immunodeficiency virus-infected ART-suppressed macaques, dual therapy with the IL-15 super-agonist N-803 and bNAbs triggered transient viremia that reprogrammed CD8^+^ T cells, achieving sustainable viral remission in approximately 70% of animals, despite only modest reductions in the viral reservoir 107. These findings underscore that enhanced immune responses, rather than complete reservoir eradication, can sustain post-ART viral control, highlighting the translational potential of immune-based HIV remission strategies in clinical practice. Recent clinical trials have provided additional evidence that bNAbs can delay viral rebound and allow partial virologic control without ART in humans. In the phase 2a TITAN trial, the administration of bNAbs (3BNC117 and 10-1074) delayed viral rebound upon ART interruption, with 36% of participants maintaining partial or complete control over 25 weeks despite subtherapeutic antibody levels 108. The antibody-mediated prevention study further demonstrated that among Southern African women who received VRC01 or placebo near HIV acquisition, 18% maintained ART-free viral suppression for ≥32 weeks, with one VRC01 recipient maintaining viral loads below 200 copies/ml for nearly two years 109. These findings imply that the early administration of bNAbs, especially alongside ART, may improve post-treatment viral control in patients with HIV. Recently, a phase 1/2a trial of a triple-bNAb combination (PGT121, PGDM1400, VRC07-523LS) in PLWH demonstrated that 83% of participants sustained virologic suppression for at least 28 weeks, with 42% achieving prolonged control for 38-44 weeks, even after antibody levels decreased 110. This long-term control is associated with reduced immune activation, T cell exhaustion, and proinflammatory signaling 110. These results suggest that triple-bNAb therapy may offer more durable ART-free viral suppression than dual-bNAb therapies. However, the effectiveness of bNAbs is limited by several factors, as HIV can mutate and evade even the most broadly acting antibodies, leading to resistant strains and subsequent rapid viral escape 99, 111. Moreover, not all PLWH respond favorably to bNAbs, and the antibodies typically require repeated administration to maintain their effect, further rendering them ineffective as a cure for HIV 99.

#### 2.1.5 Chimeric antigen receptor (CAR) T-cell therapies

Chimeric antigen receptor (CAR) T-cell therapy, originally developed for cancer treatment, has been adapted as a potential strategy for curing HIV by engineering T cells to recognize and kill HIV-infected cells 112. These modified T cells target HIV-1-specific antigens or conserved parts of the virus, such as envelope proteins, in the infected cells 113. Anti-HIV-1-engineered CAR T-cells have shown promising results, demonstrating high specificity for infected T-cells, suppression of viral replication, and delayed viral rebound 114-116. However, CAR T-cells are unable to fully reach viral reservoirs in deeper tissues 117-120. The only successful CAR T-cell model was developed in macaques treated with hematopoietic stem cell (HSC)-derived CAR T-cells 117, 120. These cells proliferate within lymphoid germinal centers and are detectable in specific brain regions, including the hippocampus and parietal cortex, in most macaques. Nevertheless, their trafficking to other parts of the CNS, such as the basal ganglia, thalamus, and cerebellum, is limited and inconsistent 117, 120, 121. A major challenge is that HIV rapidly mutates and can downregulate or hide target antigens, such as *env* epitopes, making it harder for CAR T-cells to recognize the infected cells 117, 120, 122, 123. Additionally, persistent inflammation and T-cell exhaustion in chronic HIV infection can impair the longevity and effectiveness of CAR-T cell responses in patients with HIV. Finally, CAR T-cells may struggle to penetrate and function within sites such as the LNs and the brain, where latent HIV often resides 84, 124. However, CAR T-cell therapy has not yet achieved durable viral remission in PLWH.

#### 2.1.6 Immune checkpoint modulation

Immune checkpoint modulation offers another approach, aiming to enhance the immune system's ability to fight HIV by blocking proteins, such as programmed cell death protein 1 (PD-1) and cytotoxic T-lymphocyte-associated protein 4 (CTLA-4), which normally act as “brakes” on T-cell activity 125. The goal of inhibiting these checkpoints is to revive exhausted HIV-specific T cells, helping them to better recognize and eliminate HIV-infected cells. Although this strategy has shown promise in cancer immunotherapy, its application in HIV treatment presents several challenges. In HIV, immune checkpoint blockers only produce modest improvements in T cell function and viral control, and they also risk excessive immune activation and inflammation 95, 126, 127. Moreover, reversing T cell exhaustion may not significantly increase viral clearance from latent reservoirs, limiting progress toward an HIV-1 cure 128.

#### 2.1.7 Immune challenges for HIV-1 elimination

Despite advances in ART and other therapies, the complexity of the virus, including immune evasion, latent persistence, and rapid mutation, continues to hinder the development of a definitive cure 129. However, approaches such as LRAs, gene editing, and therapeutic vaccines show promise, although each faces unique obstacles that limit their effectiveness 129, 130. A notable example is CAR T-cell therapy, which has faced several challenges. These include HIV's ability to infect and destroy CAR T-cells themselves, difficulty in targeting hidden viral reservoirs, high mutation rates, viral escape, and cytokine release syndrome (CRS), which can cause mild, flu-like symptoms or life-threatening multi-organ failure, all linked to CAR T-cell activity. Additionally, the low expression of viral antigens on infected cells and the high manufacturing and logistical costs of CAR T-cell therapy present significant limitations 131, 132. Importantly, CAR T-cells alone have not yet prevented viral rebound after ART interruption 133. Recent advances in gene editing have opened new possibilities. Studies have shown that CRISPR-Cas9-mediated integration of anti-HIV CARs into the CCR5 locus creates HIV-resistant T cells, preventing the infection of CD4^+^ CARs and preserving their cytotoxic function. Using single-chain variable fragments from bNAb 10-1074, these CAR T-cells specifically eliminated HIV-infected cells *in vitro* and temporarily limited infection in peripheral blood mononuclear cell-derived humanized mice 134. This approach also demonstrated functional expansion *in vivo*, highlighting the potential of CCR5-disrupted CAR T cells to improve therapeutic outcomes. Continued research, new combination therapies, and further extended development of CAR T-cells are essential to overcome these barriers and move toward a functional HIV cure.

### 2.2 Gene editing therapies

Gene editing has become a transformative tool in modern biology, enabling the precise manipulation of genetic material in living organisms 135. These strategies leverage the body's natural DNA repair pathways to introduce targeted insertions, deletions, or sequence replacements into the host's genome. Early methods, such as zinc-finger nucleases (ZFNs) and transcription activator-like effector nucleases (TALENs), proved the feasibility of engineered nucleases for site-specific genome modification; however, they are limited by complex protein design and low scalability 136. The emergence of the CRISPR-Cas system, adapted from a bacterial immune mechanism, has revolutionized genome engineering by providing an easily programmable, cost-effective, and versatile platform with broad applications across model organisms and in clinical settings 137. Recent studies have developed a modified CRISPR enzyme, CasX2^Max^, which carries three amino acid substitutions that enhance the sgRNA-DNA binding and catalytic efficiency in HEK293-FT cells. These modifications make CasX2^Max^ a promising tool for precise CCR5 gene disruption 138.

#### 2.2.1 ZFNs and TALENs

ZFNs and TALENs are considered first-generation gene-editing tools 136. ZFNs are chimeric nucleases created by combining a zinc-finger DNA-binding domain with the nuclease domain of an FokI-type type IIS restriction endonuclease 139. Each ZFN binds specifically to a set of three base pairs and can be combined to recognize longer DNA sequences, thereby enhancing the specificity and effectiveness of gene targeting 140. Another essential part of ZFNs is the restriction endonuclease domain, which facilitates DNA cleavage 140. This feature enables ZFNs to be re-engineered to target specific sites within the human genome 141. Several studies have shown that ZFN-mediated disruption of the CXCR4 gene in CD4^+^ T cells confers resistance to HIV 142, 143. Compared to lentivirus-delivered CXCR4-targeting short hairpin RNAs, ZFN-modified cells displayed sustained enrichment and reduced HIV-1 viral loads *in vitro* and *in vivo*, indicating that CXCR4 modification is a promising genetic approach for HIV resistance 143. ZFNs have also been used to simultaneously disrupt CCR5 and CXCR4 genes in primary human CD4^+^ T cells, resulting in normal proliferation and strong resistance to both CCR5- and CXCR4-tropic HIV-1 strains *in vitro* 142. However, in a humanized mouse model, these gene-modified cells were successfully engrafted and trafficked normally, but resistance to HIV-1 infection was only temporarily observed *in vivo* 142.

TALENs are another class of naturally derived gene-editing tools similar to ZFNs that have demonstrated efficacy in editing host genes, such as CCR5 and CXCR4, the proviral integration-associated protein LEDGF/p75, and long terminal repeat (LTR) regions of HIV proviral DNA 144, 145. Preclinical studies have provided proof-of-concept that TALEN proteins can be effectively encapsulated within a nanocapsule-based delivery system for efficient gene editing (*ex vivo*) in primary HIV-1 reservoir cells, such as T cells and macrophages 146. The authors adopted an *in situ* polymerization technique to wrap TALEN proteins encapsulated in cationic polymeric shells, specifically targeting the HIV-1 transcription activator-responsive region 146. The effectiveness of HIV-1 gene editing using this nanocapsule was further confirmed by the observed reduction in HIV-1 p24 expression in latently infected T cells 146. However, the inherent complexity and large size of TALENs, along with difficulties related to their efficient packaging, delivery, and scalability, continue to pose significant challenges for successful clinical translation 136, 144, 145.

Although these early preclinical tests demonstrated the possibility of modifying CXCR4 and/or CCR5 to prevent or treat HIV infections, the labor-intensive nature of protein engineering and limited scalability have restricted their broad application 144. These obstacles have been addressed through the development and adoption of CRISPR-Cas systems, which are natural bacterial defense mechanisms that have become transformative genome-editing tools owing to their simplicity, versatility, and high specificity 135, 147. CRISPR technologies have shown promise in preclinical animal models by targeting either the HIV genome or essential co-receptors, such as CCR5 and CXCR4 38, 148. Advances in materials science and chemical engineering have facilitated the development of versatile and more effective delivery platforms, offering improved precision and safety for CRISPR-based therapies compared with earlier generation tools 149, 150.

#### 2.2.2 CRISPR-based HIV-1 cure strategies

**Figure 5:**
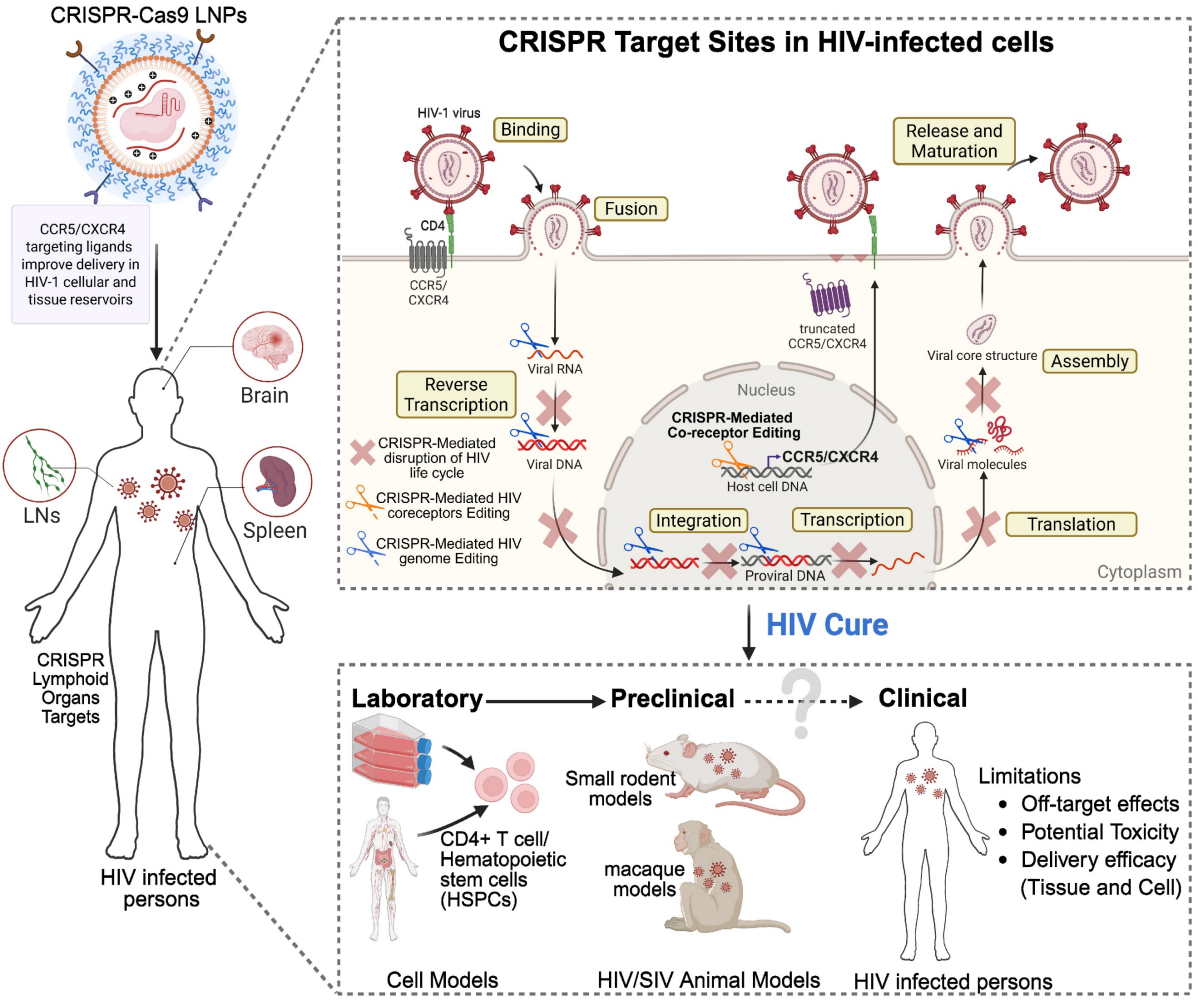
** Strategic HIV elimination targeting cellular and tissue reservoirs using CRISPR-Cas9.** CRISPR-Cas9-based systems provide high specificity for latent proviral DNA and represent a promising approach for eliminating viral reservoirs. In this system, viral guide RNAs (gRNAs) recognize complementary sequences in latent HIV DNA, which are then excised by Cas enzymes. Beyond targeting viral DNA, CRISPR-Cas9 can also be used to disrupt host genes (for example, CCR5 or CXCR4) to block co-receptor binding, inhibit viral entry, and suppress replication. CRISPR-Cas9 technology can intervene at multiple stages of the HIV life cycle, including reverse transcription, integration, viral protein transcription, and assembly, thereby disrupting viral core formation and replication. Despite its genetic specificity, delivery to infected cells remains a major challenge. Viral vectors (e.g., adeno-associated viruses (AAVs)) or LNPs are commonly used; however, optimizing efficient delivery to all reservoir cells is ongoing. To better target immune cells within viral reservoirs, strategies to incorporate ligands for immune cell receptors, such as for CCR5 and/or CXCR4, into the delivery platforms are anticipated to improve target specificity and delivery efficiency. Preclinical testing of targeted CRISPR-Cas9 delivery systems is currently undergoing in laboratory settings, including HIV-infected CD4^+^ T cells and macrophages, small rodent models, and SHIV-infected macaques, with eventual translation to HIV-1-infected subjects. While the delivery and excision specificities of CRISPR-Cas9 systems are promising, potential limitations that may affect treatment outcomes include off-target editing of the host genome, inefficient delivery to latent reservoirs, endosomal degradation of LNP cargo, immunogenicity to bacterial CRISPR-Cas9 components, emergence of HIV escape variants, and possible toxicities. Created with BioRender.com.

Early CRISPR efforts primarily targeted the viral genome, often LTRs or essential genes, to excise or inactivate the provirus 151-154. However, incomplete editing, rapid viral escape, and delivery constraints limit the durability of viral inactivation 155. Consequently, the focus has shifted to genetically stable host entry factors, especially CCR5 and CXCR4, and combination treatments that combine co-receptor editing with ART and/or proviral removal 156. Although initial studies targeted the provirus directly 143, 157, new evidence indicates that long-term HIV control requires both the prevention of new infections and the depletion of viral reservoirs 158. This has led to a strategic shift toward co-receptor editing and multimodal therapeutic approaches. Building on these findings, current research more often combines CRISPR disruption of CCR5 and CXCR4 with long-acting ART or proviral removal to block viral spread and progressively diminish latent reservoirs (**Figures 4 and 5; Table 1**) 159, 160. Screening for CRISPR editing success is typically performed on CD4^+^ T cells and HSCs. One of the earliest studies demonstrated the superior efficiency of CCR5 editing compared with previous methods 161. Using lentiviral vectors that express Cas9 and single-guide RNAs (sgRNAs) targeting CCR5, a single transduction into HIV-1-susceptible human CD4^+^ cells resulted in significant CCR5 gene disruption, with approximately 43% of the cells exhibiting CCR5 disruption. These CCR5-disrupted cells were resistant to R5-tropic HIV-1 and had a selective advantage over unmodified cells during infection 161. Additionally, a T7 endonuclease I assay used to check for off-target mutations in stably transduced cells found no off-target effects. Similarly, researchers have explored whether adenovirus-delivered CRISPR-Cas9 can effectively edit CCR5 in primary human CD4^+^ T cells 162. They isolated cells from donors, delivered the CRISPR-Cas9 system via adenoviral vectors, and tested the susceptibility of the cells to HIV infection. They successfully disrupted CCR5 in 74.1 and 63.8% of primary T cells from the two donors, respectively. These edited cells also showed resistance to CCR5-tropic HIV-1, with resistance levels similar to those reported in previous studies (**Table 1**) 162. To further examine CCR5 as a therapeutic target, Wang *et al.* studied whether targeting CCR5 in human induced pluripotent stem cells (iPSCs) using CRISPR could create immune cells resistant to CCR5-tropic HIV-1 163. Editing frequencies reached 27% with single guide RNAs and 41% with dual guide RNAs for biallelic disruption, respectively. These results indicate that homozygous CCR5 mutations confer resistance to CCR5-tropic HIV-1, suggesting that iPSC-derived CCR5-disrupted cells could be a source of HIV-resistant immune cells. To further investigate the role of HIV-1 co-receptors, CCR5 and CXCR4 genes were simultaneously disrupted in human CD4^+^ T cell lines using CRISPR-Cas9, achieving editing efficiencies of 55 and 36%, respectively 164. The edited cells showed resistance to both HIV-1_NL4-3_ (CXCR4-tropic) and HIV-1_YU-2_ (CCR5-tropic) strains 164. Moreover, studies have indicated no significant difference in apoptosis between gene-edited and unmodified cells based on flow cytometry with 7-AAD and annexin V, which assess cellular death and apoptosis, respectively 165. Deep sequencing and T7E1 assays of the predicted off-target sites revealed no detectable off-target effects. A recent study focused on disrupting CXCR4 and CCR5 by introducing stop codons using base editing in primary human CD4^+^ T cells 39. Flow cytometry showed that CXCR4 expression decreased by 75 and 86% on day 4, whereas each guide RNA achieved an 80-94% reduction in CCR5 expression by day 11. To evaluate T cell function, cytokine production (IL-2, IL-4, IFN-γ, and TNF-α) was measured in both control and dual-receptor-edited T cells, which showed similar levels of cytokine production, indicating that these cells retained their functional abilities. Finally, the ability of co-receptor knockout cells to resist HIV infection was tested using replication-incompetent lentivirus pseudotypes with CCR5- or CXCR4-tropic HIV-1 envelopes. The edited cells exhibited a significant reduction in co-receptor expression and viral infection 39.

Building on the success of early studies, CRISPR-based strategies have been developed for animal studies 38, 166-168. A single CRISPR-Cas9 editing system was used to disrupt CCR5 in human CD34^+^ HSCs (**Table 1**) 169. The edited HSCs were transplanted into humanized NOD.Cg-Prkdc^scid^ll2rg^tm1Wjl^/SzJ (NSG) mice, which were subsequently challenged with HIV-1. Efficient and durable CCR5 excision in T cells from long-term HSCs was observed, and mice that received edited cells exhibited significantly reduced HIV-1 RNA levels. This study demonstrated that CRISPR-Cas9-mediated CCR5 disruption in long-term repopulating HSCs is both feasible and effective, supporting the potential of CCR5-edited HSCs as a therapeutic strategy for HIV-1 169. A similar study was conducted targeting the CXCR4 receptor using CRISPR-Cas9 in primary human CD4^+^ T cells 159, in which four gRNAs that efficiently induced indel mutations in the CXCR4 gene were identified. Following lentiviral transduction with CXCR4-targeting gRNAs, the proportion of CXCR4-expressing cells decreased by 70-77%. To assess the protective effect of CXCR4 disruption, the cells were challenged with a CXCR4-tropic HIV-1 strain. In CXCR4-edited cells, viral expression was completely absent, whereas 55% of the infected control cells showed detectable viral activity 159. Another study identified four gRNAs that efficiently induced high-frequency editing of the human CCR5 gene 170. When introduced into primary T cells, all four gRNAs successfully disrupted CCR5, leading to a significant reduction in the proportion of CD4^+^ and CD8^+^ T cells expressing surface CCR5. To assess the impact on HIV susceptibility, stimulated human PBMCs were electroporated with Cas9 and either CCR5-specific gRNAs or a control GFP-specific gRNA and then challenged with a high dose of CCR5-tropic HIV-1JR-CSF. CCR5-edited CD4^+^ T cells showed a sustained reduction in HIV infection compared to those treated with GFP gRNA. Importantly, the degree of infection resistance correlates with a decrease in CCR5^+^ CD4^+^ T-cells, supporting CCR5 editing as an effective strategy to confer resistance to HIV in human T-cells 170. A preclinical study was conducted to confer HIV resistance by simultaneously disrupting CCR5 and CXCR4 38. Successfully edited T lymphocytes reduce the surface expression of both co-receptors, resulting in resistance to R5, X4, and dual-tropic HIV-1 strains. In addition, disrupted CCR5 alleles in human CD34^+^ hematopoietic stem and progenitor cells (HSPCs) generate HIV-resistant macrophages. In humanized mice, HSPCs with dual co-receptor disruption showed poor engraftment in the bone marrow and reconstitution of CD4^+^ T cells, whereas CXCR4-disrupted CD4^+^ T cells were enriched in the peripheral blood and spleen after the HIV-1 challenge. These results likely reflect the survival advantage conferred by resistance to viral infections 38.

#### 2.2.3 Immune and Safety Implications for CCR5 and CXCR4 Disruption

Although modifying both CXCR4 and CCR5 receptors is promising for conferring broad resistance to HIV-1, it raises important concerns because of their fundamental roles in immune system regulation. CXCR4 is critical for HSC homing, retention, and migration within the bone marrow niche and plays a key role in thymic T cell development and lymphoid tissue organization 171. Therefore, disruption of CXCR4 function may impair hematopoiesis, reduce thymic output, and disturb the normal immune cell distribution. CCR5, on the other hand, is essential for the recruitment and activation of immune cells, such as T cells and macrophages, during inflammatory and infectious responses 172. Loss of this receptor may lead to altered immune surveillance, diminished responsiveness to pathogens, and dysregulated cytokine signaling. Consequently, the simultaneous disruption of both CXCR4 and CCR5 can compromise immune homeostasis and weaken host defenses against infection or vaccination. Therefore, long-term risk management is essential, as such interventions may increase susceptibility to opportunistic infections, cause immune dysregulation, impair CAR-T cell trafficking to target sites, and lead to other immune-related complications. Although short-term *in vitro* studies have demonstrated that dual-edited cells remain viable and functionally competent, the long-term immunological consequences remain insufficiently characterized and require comprehensive evaluation before clinical application 165. The CXCR4/CXCL12 and CCR5/CCL5 signaling axes are key mediators of crosstalk between malignant cells and the tumor microenvironment, playing critical roles in the migration and metastasis of epithelial malignancies 173. Building on this understanding, preclinical studies have demonstrated the therapeutic efficacy of CXCR4 and CCR5 antagonists in limiting tumor progression 174, 175. Although the long-term oncological effects of HIV-1 co-receptor editing remain largely unexplored, the therapeutic repositioning of HIV drugs targeting these receptors is being investigated in oncology. This approach is based on the rationale that disrupting CXCR4 and CCR5 functions can suppress the migratory and metastatic potential of malignant tumors, offering a promising avenue for anticancer therapy 176.

## 3. Combinatorial therapies for viral elimination

Combinatorial approaches that simultaneously target viral co-receptors and other critical stages of the HIV life cycle and replication have gained increasing attention because of their improved therapeutic efficacy compared with monotherapies 177-179. Recent CRISPR-Cas9-based gene therapies, such as CCR5 gene editing and proviral excision, have shown considerable promise but have also highlighted persistent challenges related to *in vivo* delivery, editing efficiency, and the long-term persistence of edited cells 178.

Recent studies have explored multimodal strategies to overcome these limitations. In this context, Herskovitz *et al.* reviewed the application of CRISPR-Cas9 to eliminate latent HIV-1, highlighting its potential and addressing critical challenges related to delivery, precision, and safety 180. Building on this, they demonstrated that CRISPR-Cas9 mRNA combined with TatDE gRNAs and delivered with lipid nanoparticles (LNPs) efficiently and specifically disrupts multiple HIV-1 exons, enabling the excision of latent proviral DNA across diverse viral strains and underscoring a promising gene-editing strategy for HIV-1 eradication 181. A recent study treated HIV-1-infected humanized NSG mice with a combination of long-acting ester ART prodrugs and CRISPR-Cas9-mediated gene-editing constructs targeting both CCR5 and HIV-1 proviral DNA sequences 148. CCR5 gene editing targets specific regions, including CCR5Δ32 deletion, which yields cells lacking CCR5 expression and are resistant to infection by CCR5-tropic HIV-1 148. Following viral infection, the mice underwent a three-step treatment protocol that included a long-acting, slow-effective release ART (LASER-ART) regimen comprising nanoformulated myristoylated cabotegravir, lamivudine, abacavir, and native rilpivirine. This regimen suppressed viral replication in conjunction with CCR5 gene editing using CRISPR-Cas9. The subsequent step involved the use of AAV9-CRISPR-Cas9 to remove the targeted proviral DNA sequences, specifically by cleaving the LTR-Gag region. Notably, 58% of the animals in the ART and dual CRISPR treatment groups displayed undetectable plasma viral RNA levels. Results from reservoir tissues indicated that the combinatorial strategy, including LASER-ART, CCR5 gene editing, and HIV-1 CRISPR, was more effective than separate treatments in reducing viral DNA levels 148.

Similarly, a dual gene-editing approach was developed to create cells resistant to both CCR5-tropic and CXCR4-tropic HIV-1 strains 182. This method involved using CRISPR-Cas9 to knockout CCR5 while expressing C46, a membrane-bound HIV-1 fusion inhibitor, in the cells. In a human T-cell line model, cells showed significantly reduced HIV-1 replication and increased survival after exposure to CCR5-tropic or CXCR4-tropic HIV-1 strains 182. Additionally, rilpivirine-loaded LNPs functionalized with a CCR5 ligand successfully targeted myeloid cell reservoirs and crossed the blood-brain barrier, highlighting the potential of CCR5-targeted delivery systems for CRISPR-Cas9-based HIV therapy 183. Recently, our group has developed CXCR4 ligand-functionalized LNPs designed for the targeted delivery of CRISPR-Cas9 and gRNAs to eliminate latent HIV-1 from CXCR4-expressing cells 184. These LNPs improved mRNA translation and cellular uptake through CXCR4-mediated mechanisms, enabling the efficient removal of HIV-1 DNA from infected CD4^+^ T cells. We used two lymphocytic cell lines with high CXCR4 expression to demonstrate the cell-specific mRNA delivery. Recognizing the challenges of transfecting CD4^+^ T cells, this study focused on creating cell-selective CXCR4-targeted LNPs (T-LNPs) to ensure the effective delivery of CRISPR-m1Ψ-mCas9-gRNA for LNP uptake and subsequent HIV-1 DNA excision within T cells. The use of targeted LNPs resulted in an 80% overall excision of HIV-1 DNA in lymphocytes after multiple doses. In humanized mouse models, the combination of ART with receptor-targeted LNPs resulted in approximately 60% excision efficiency in the blood and spleen tissues. This underscores the potential of targeted LNPs to specifically target and reduce HIV-1 proviral DNA in latently infected and ART-suppressed lymphoblasts (**Figure 5**) 184. Notably, to explore CXCR4 as a therapeutic target for HIV, a naturally occurring human CXCR4 mutant, P191A, was generated using a combination of CRISPR and PiggyBac transposon technologies 185. Treatment with either two distinct gRNAs or a duplex CRISPR system resulted in a significant reduction in the CXCR4^+^ targeted cell population from 99.8 to 18.4%, 12.0%, and 11.6%, respectively. DNA sequencing confirmed a homozygous P191A mutation. When these cells were infected with HIV-1_NL4-3,_ a 50% decrease in HIV-1 replication was observed compared to that in wild-type cells, indicating that the P191A mutation confers resistance to HIV-1. Although these approaches demonstrate strong viral suppression and elimination of latent proviral DNA, their greatest potential may lie in thoughtfully designed combinations that incorporate newly developed ultra-long-acting ART (ULA-ART), latency reversal, immune clearance, and gene editing. For example, the “shock-and-kill” strategy reactivates latent viruses using LRAs, allowing for immune clearance or targeted gene editing. When combined with CRISPR, this could increase Cas9 access to reactivated proviruses, thereby boosting editing efficiency.

Conversely, in a “block-and-lock” model aimed at deepening latency, CRISPR could be redundant unless it is used for permanent silencing or excision of the target gene. Another promising approach is the combination of bNAbs and CRISPR systems. bNAbs may help eliminate reactivated infected cells or prevent reinfection after CRISPR-based proviral DNA excision, potentially serving both therapeutic and prophylactic roles. However, the timing, immune engagement, and potential immunogenicity of CRISPR components must be carefully optimized to avoid redundancy or adverse effects. **Table 2** compares the different HIV cure strategies used. It focuses on combinations such as CRISPR with ART, immunotherapy, LRAs, bNAbs, and engineered T cells. The table shows how these strategies work together. It also lists their benefits, drawbacks, research progress, and potential risks. Overall, although no single combination has yet achieved consistent HIV eradication, available evidence indicates that the combination of CRISPR with pharmacological and immunological approaches provides synergistic benefits in HIV treatment. Future improvements should focus on enhancing delivery methods, reducing immunogenicity, and customizing the regimen timing to maximize synergy and prevent redundancy. Such thoughtfully designed combinations are the most promising path toward sterilizing or functional HIV-1 cures.

## 4. Lab-to-clinic pathways to achieve an HIV cure

Although CCR5 gene editing has shown significant promise in *in vitro* studies and animal models, its clinical application in PLWH has been limited to a few isolated cases and has not yet been broadly adopted 186. Several clinical trials have been conducted based on various HIV-1 cure strategies that primarily aim to achieve substantial viral suppression with ART while targeting different aspects of HIV-1 pathogenesis.

These approaches mainly include the eradication of multiple viral reservoirs across anatomical and cellular compartments, attaining strong control of viral replication through immune-enhancing, and combination therapies that synergistically integrate with immune-boosting and reservoir-eliminating modalities 187. One of the first trials to assess CCR5 gene editing in humans employed ZFN-mediated modification of autologous CD4^+^ T cells (NCT03666871) 188. In a non-randomized study involving 12 HIV-1 infected individuals on long-term ART, CCR5-edited cells constituted approximately 11-28% of the total circulating CD4⁺ T cells one-week post-infusion. Edited cell frequencies gradually declined, with an estimated half-life of 48 weeks, but persisted for up to 42 months. All participants experienced viral rebound approximately 2-4 weeks after starting ATI. A single grade 3 transfusion-related adverse event (fever, chills, or arthralgia) was observed, while the remaining adverse events were mild to moderate and transient. The presence of the characteristic five-nucleotide “pentamer” duplication at the ZFN target locus, detected by PCR-based assays, confirmed on-target editing with minimal off-target modifications 188. A subsequent study evaluated ZFN-modified hematopoietic stem and progenitor cells (HSPCs) for sustained CCR5 disruption (NCT02500849) (**Table 1**) 189, 190. Gene editing outcomes were verified using multiple orthogonal assays, including PCR amplification of the target site, surveyor nuclease mismatch detection, and targeted genome sequencing to confirm the disruption of CCR5 with high specificity. The editing efficiency ranged between 50-60% in the infused HSPC population. No severe or dose-limiting adverse events were reported; mild injection-site reactions and transient cytokine elevations (grade 1-2) resolved spontaneously. Depending on the cohort, follow-up ranged from 4 to 24 weeks. Viral rebound occurred at 2-4 weeks post-ATI, similar to that a in previous study.

The critical role of CCR5 in the HIV-1 life cycle and preclinical reports suggest that CCR5 antagonism can avert viral entry, which has led to the therapeutic development of strategies that can mutate CCR5 *ex vivo,* which can subsequently be administered to PLWH to achieve immune control and viral elimination 191-193. In China, a clinical trial (NCT03164135) was conducted that recapitulated a similar clinical strategy that yielded a functional cure for HIV-1 in the London and Berlin patients. This trial employed CRISPR/Cas9-edited CCR5-ablated HSPCs that were transplanted into an HIV-1 infected individual with acute lymphoblastic leukemia (**Table 1**) 194. The patient achieved complete donor chimerism, and long-term follow-up (~19 months) demonstrated stable engraftment of CCR5-disrupted cells (17.8% *in vitro* editing; 5.2-8.3% *in vivo* CCR5 disruption). Adverse events were limited to preconditioning-related toxicities, such as anemia, neutropenia, and thrombocytopenia. Viral rebound was observed approximately four weeks after ATI, prompting ART resumption. Gene disruption was confirmed by targeted deep sequencing, which demonstrated precise CRISPR-mediated cleavage with minimal off-target activity. The investigators concluded that the response of transplanted CD4^+^ T cells was insufficient for an HIV-1 cure but demonstrated that the long-term engraftment of CCR5-ablated HSPCs could sustain continuous peripheral cell production 194. However, beyond technical challenges, ethical considerations add another layer of complexity to the widespread application of CCR5 editing 195.

Recently, a clinical trial of the investigational gene therapy EBT-101 (NCT05144386) evaluated a CRISPR-based dual-excision strategy delivered via an adeno-associated virus vector serotype 9 (AAV9) vector 196, 197. This single ascending-dose, open-label study included six participants receiving stable ART. Treatment-emergent adverse events were predominantly grade 1 and 2 in severity, including transient complement activation, with no serious or dose-limiting toxicities. The median viral rebound occurred at 3-4 weeks post-ATI, although one participant maintained viral suppression for up to 16 weeks. The primary study follow-up is ongoing for 48 weeks, with long-term monitoring planned for up to 15 years to assess durability and safety. Viral reservoir changes were evaluated using an intact proviral DNA assay. The initial analysis found no evidence of off-target DNA cleavage, supporting the development of nucleic acid-based therapies for diverse populations. Another crucial strategy in the pursuit of a functional HIV-1 cure is the “shock-and-kill” approach, which was tested in a clinical study using LRAs to examine the combined effect of vorinostat with AGS-004 in five HIV-1 infected individuals on stable ART (NCT01069809) 188. This study measured multiple parameters indicative of viral replication, including HIV-specific T cell responses and resting CD4^+^ T cell-associated viral RNA (rca-RNA) levels, after therapy. The goal was to combine vorinostat's latency reversal with HIV-specific immune induction through AGS-004; however, the therapeutic intervention had no significant impact on the pre-existing reservoir size, as assessed by a quantitative viral outgrowth assay (QVOA). Two out of five participants showed a decrease in rca-RNA, but this did not correlate with the infection levels in resting CD4^+^ T cells. The researchers attributed these results to the depletion of replication-incompetent cells and limited longitudinal data on rca-RNA 198. Collectively, these early clinical investigations demonstrate that gene-editing strategies targeting CCR5 and integrated proviral DNA can be performed safely, with consistent *in vivo* persistence of edited cells and manageable adverse event profiles. However, the relatively short median time to viral rebound post-ATI underscores the need for higher engraftment efficiencies, more comprehensive reservoir targeting, including lymphoid tissues, and refined assays to confirm the longitudinal editing efficiency. Future studies integrating quantitative measures of engraftment, durability, and functional viral control are essential for advancing the translational potential of genome editing-based HIV-1 cure strategies (**Figure 5**).

## 5. Social and ethical considerations

Using stem cell transplantation in otherwise healthy individuals with well-controlled HIV infection on ART is ethically questionable because of the potential for graft-versus-host disease and transplant-related mortality, which together pose a significant risk 189. Similarly, germline CCR5 editing in embryos has raised significant societal and regulatory concerns because of the potential for serious injury, disability, and misapplication if used without proper surveillance 199, 200. For instance, in the first reported case of gene-edited babies, the scientist who used CRISPR-Cas9 to edit embryos was warned to conduct illegal medical procedures 201. Recent ethical discussions have concluded that while safety remains the primary concern in debates over heritable genome editing, meaningful public engagement must also address deeper categorical and sociopolitical issues that reflect broader societal values 202, 203. These issues highlight persistent barriers, including ethical dilemmas, safety risks, procedural challenges, and inadequate editing efficiency, which continue to hinder the widespread implementation of CCR5 editing strategies.

Recent discussions have emphasized that monotherapies are unlikely to provide lasting remission, increasing the interest in combination HIV cure strategies 204. Approaches combining LRAs with immune-based interventions have strong preclinical and early clinical data but also raise additional safety and ethical issues 205-207. Many LRAs, especially repurposed cancer drugs, have been linked to mutagenicity, off-target genetic effects, and blood-related toxicities, while their capacity to significantly reduce the latent reservoir remains unproven 206-208. The participants in these studies warned that the widespread biological disruptions caused by latency reversal require personalized assessments, strict eligibility criteria, and long-term monitoring to minimize harm 206, 208. Additionally, immune-based agents pose unique risks, making it difficult to ensure a favorable benefit-to-risk ratio for otherwise healthy individuals living with HIV 208, 209. Another important factor is whether these strategies offer meaningful improvements over standard ART, which is safe, effective, and broadly accessible to patients. Stakeholders stressed that the expected benefits of any cure regimen must outweigh the risks and align with other priorities, such as reducing the treatment burden, enhancing the quality of life, and ensuring fair access 208. Community involvement is crucial for defining relevant outcomes, managing expectations, and addressing stigma- and cost-related issues 208, 210. Notably, structural barriers, such as limited industry incentives for collaboration and the absence of standardized trial endpoints, have been identified as obstacles to advancing combination HIV cure strategies 208. Overall, both CCR5-targeted approaches and LRA immune-based combinations highlight the scientific and ethical challenges in advancing HIV cure research. To sustain trust and uphold ethical standards, clinical trial designs must include rigorous safety monitoring, early regulatory engagement, and continuous community input 205, 211. Ultimately, balancing innovation with participant protection is crucial for transforming these investigational approaches into safe, scalable, and socially acceptable treatment options.

## 6. Conclusions and future perspectives

Targeting the HIV co-receptors CCR5 and CXCR4 and using lymphoid-targeting LNP approaches marks a major advancement in HIV therapy, especially in tackling the challenge of latent viral reservoirs 186. While ART effectively suppresses HIV replication, these reservoirs are not eradicated, leading to viral rebound once treatment is stopped 186, 212.

Previous strategies, such as those using LRAs and bNAbs, have failed to eliminate reservoirs and have raised additional safety concerns, highlighting the need for more innovative solutions 213. Next-generation CRISPR-Cas systems are promising tools for the precise editing of host co-receptors CCR5 and CXCR4, which are essential for HIV entry. These have shown potential in creating CD4^+^ T cells and HSCs resistant to both R5- and X4-tropic HIV strains, significantly decreasing viral reservoir sizes in infected animal models 38. These results emphasize the therapeutic potential of co-receptor targeting to achieve sustained HIV suppression. However, the efficient delivery of CRISPR-Cas components remains a key challenge in therapeutic genome editing, with both viral and non-viral platforms actively explored for their potential to maximize editing efficiency while minimizing toxicity and off-target effects of treatment. Viral vectors, particularly AAV and lentiviruses, have been widely utilized because of their high transduction efficiency and ability to reach diverse cell types. AAV vectors are currently favored for *in vivo* applications because of their broad tissue tropism, low immunogenicity, and non-integrating nature, which contribute to their enhanced biosafety profiles 148, 158, 214. However, the limited packaging capacity of AAVs (4.7-5.0 kb) limits their broad use when delivering large Cas9 systems with sgRNA 215, and pre-existing neutralizing antibodies can decrease transduction efficiency in humans 215, 216. Lentiviral vectors enable stable gene integration and support delivery into non-dividing cells, with a high cargo capacity and the ability to target specific cell types through pseudotyping 181, 214, 217. However, concerns regarding insertional mutagenesis have restricted their clinical use 181, 218. Non-viral delivery systems, which are broadly categorized into physical and chemical approaches, offer modular and safer alternatives. Physical methods, such as electroporation, microinjection, and ultrasound-mediated delivery, facilitate direct cytosolic access but often damage cellular integrity, limiting their use to *ex vivo* and *in vitro* studies 219. Chemical approaches, such as polymeric or lipid-based nanoparticles and liposomes, offer several advantages, including lower immunogenicity, reduced toxicity, and the ability to be surface-functionalized with targeting ligands for cell-specific delivery 184, 220-222. Their cost-effectiveness, high cargo-loading capacity, and favorable biocompatibility make them promising candidates for next-generation gene delivery systems. However, challenges such as degradation during circulation, endosomal entrapment, and limited nuclear delivery in non-dividing cells must be addressed to fully realize their clinical potential 223, 224. Ongoing research on advanced delivery platforms, rapid excision, and in situ mutation detection assays 146, 225 may further strengthen the efficacy and safety of these approaches by enabling real-time monitoring of genetic alterations in edited cells.

In summary, immune-based therapies, gene-editing technologies, and combinatorial approaches for viral elimination have shown promising outcomes in preclinical studies and are progressing through various stages of clinical evaluation. However, despite these advances, a definitive cure for HIV-1 remains elusive. Among these challenges, disruption of CXCR4 and CCR5 co-receptors through CRISPR-Cas9 editing presents potential long-term safety risks, including increased susceptibility to opportunistic infections, immune dysregulation, and impaired CAR-T cell trafficking. Therefore, a deeper understanding of HIV-1 pathogenesis, combined with careful management of safety concerns through optimized delivery systems and refined CRISPR technologies, would be essential for developing safer, more effective, and durable strategies toward a functional HIV-1 cure.

## Figures and Tables

**Table 1:** CXCR4 and CCR5 receptor gene editing and HIV-1 elimination

Year	CRISPR Modifications	Duration or Dose	Outcomes	Ref.
2015	Adenovirus delivery of CRISPR-Cas9 with sgRNAs to disrupt CCR5 in primary CD4^+^ T cells and conferring HIV-1 resistance.	CCR5 gene editing in primary CD4^+^ T cells was achieved using the Ad5F35 adenoviral vector at a MOI of 30 or 100, with maximal editing at 8 days.	Up to 74% indels, effectively eliminating CCR5 in primary CD4⁺ T cells. This confers resistance to HIV-1 infection.	162
2015	CRISPR/Cas9-mediated disruption (knockout) of the CXCR4 gene in human primary CD4⁺ T cells to block HIV-1 entry and create HIV-resistant immune cells.	IL-2 treated primary human CD4⁺ T cells were stimulated with anti-CD3/CD28 for 2-3 days, electroporated with 2 μg CXCR4-gRNA-Cas9 plasmid/5×10^6^ cells, cultured for 2 days, then assessed after 6 to 8 hours HIV-1 exposure.	CRISPR/Cas9 disruption of CXCR4 in primary human CD4^+^ T cells reduced HIV-1 infection by >64% at day 7 compared to control cells.	159
2017	Targeted knock out of CCR5 gene in human CD34^+^ hematopoietic stem/progenitor cells.	Analysis of human hematopoietic reconstitution and CCR5 disruption in mice from 6 to 12 weeks post-transplantation, with key evaluations performed at week 12.	CCR5-disrupted mice maintained 94% of their CD4^+^ T cells compared to 37% in controls.	169
2019	CRISPR-edited CCR5-ablated HSPCs and progenitor cells were transplanted into an HIV-1 infected patient.	The patient received a single infusion of CRISPR-edited CD34^+^ along with unedited cells after myeloablative (cyclophosphamide and total-body irradiation) conditioning. Follow-up included 4-weeks of ART interruption at 7 months.	CRISPR-mediated CCR5 disruption ranged from 5 to 8% in bone marrow cells over 19 months post-transplant, demonstrating long-term engraftment of edited HSPCs but with insufficient efficiency to cure HIV-1 infection.	194
2021	Disrupt the CCR5 gene in HSCs to create HIV-resistant immune cells.	Refers to generally low-dose CCR5-edited HSPC infusion.	Disrupting the CCR5 gene in HSCs aims to create HIV-resistant immune cells, inspired by the 0% increased mortality in CCR5-∆32 homozygotes and the 31.2% efficacy seen in the RV144 HIV vaccine trial.	169, 189, 190
2022	Disruption of CCR5 and CXCR4 genes in T cells and HSPCs, creating resistance to multiple HIV-1 strains.	Cas9/gRNA was given to human CD4^+^ T cells by electroporation, analyzed gene editing, and results assessed in mice up to six weeks.	CRISPR-Cas9 disruption achieved up to 88% reduction of CCR5 and CXCR4 in primary T cells, with editing efficiencies of 22-62% in HSPC and T cells. There was resistance to multiple HIV-1 strains.	38
2023	Targeted CCR5 gene and HIV-1 proviral DNA.	ART was for 4 weeks, followed by a recovery period, then CRISPR therapy.	Elimination of HIV -1 proviral DNA in lymphoid, bone marrow, and CNS tissues in 58% of infected animals.	148
2025	Knocked out CCR5 gene in human stem cells.	Mice were transplanted with 10^6^ HSPCs in five doses of CCR5 edited HSPCs. The balance of HSPCs comprising mock edited (GFP gRNA) cells.	The results showed over 90% protection against HIV infection.	170
2025	To remove latent HIV-1 proviral DNA from infected CD4^+^ T cells.	Repeated dosing of targeted LNPs carrying CRISPR.	Repeated LNP dosing in ART-treated humanized mice achieves 80% HIV-1 DNA excision.	184

**Table 2:** Combinatorial HIV-1 Cure Strategies

Strategy	Key Components	Synergy / Advantages	Risks / Limitations	Status	Ref.
**Shock-and-Kill + CRISPR**	CRISPRa or LRAs + CRISPR editing	Latency reversal increases proviral accessibility for CRISPR excision	Limited *in vivo* validation; risk of incomplete editing and off-target effects	Preclinical (cell models, proof-of-concept)	83
**LASER-ART + CRISPR**	Long-acting ART + AAV9-CRISPR targeting LTR/Gag	Dual suppression + proviral excision; reduced rebound; tissue clearance	Partial cures in mice; delivery efficiency and off-target risk	Preclinical (humanized mice)	158
**CRISPR + ART (EBT-101)**	AAV9-SaCas9 + gRNAs with suppressive ART	Multiplex targeting lowers viral escape: ART maintains suppression during editing	Viral rebound in early trial; incomplete eradication of latent reservoirs	Phase I/IIa (first-in-human)	196, 197
**Immunotherapy + LRAs**	AGS-004 dendritic vaccine + vorinostat	Safe; modest T-cell induction; partial reservoir effects	No persistent impact on replication-competent reservoir	Early clinical trial	198
**Shock-and-Kill + bNAbs**	Vorinostat + long acting bNAbs	Potential immune clearance of reactivated cells	Weak LRA potency; limited reservoir reduction	Phase I trial	226
**bNAbs + Immune Modulators**	bNAbs + IL-15 superagonist or TLR7 agonist	Enhances immune clearance; delayed viral rebound in macaques	Anti-drug antibody responses; incomplete reservoir elimination	Preclinical (SHIV models)	107
**CRISPR-Engineered CAR T Cells**	CRISPR/Cas9-mediated CCR5 disruption + CAR integration (10-1074 bNAb scFv)	CCR5 knockout confers resistance; CAR T-cells eliminate HIV-infected cells; *in vivo* expansion	Limited to R5-tropic strains; partial editing; viral escape; transient suppression	Preclinical (*in vitro*, hu-PBMC mice)	134
**CRISPR Dual Targeting**	CCR5 knockout + C46 fusion inhibitor	Broad protection against R5 and X4 HIV-1 strains	Tested only in cell lines; vector immunogenicity	Preclinical	182
**CXCR4-Targeted LNPs + CRISPR**	CXCR4 receptor and lymphoid tissue targeted LNPs + CRISPR + ART	Selective T-cell targeting; approximately 60 to 80% proviral excision	Delivery scalability; tissue variability	Preclinical (*in vitro*, hu mice)	184
**Block-and-Lock (CRISPR/dCas9-KRAB)**	dCas9 repressors targeting HIV LTRs	Persistent latency; prevents reactivation	Requires sustained expression; safety unknown	Preclinical	227
**CRISPR-Edited HSPCs**	CCR5-edited stem cells + ART	Long-term engraftment; partial immune protection	Rebound after ART interruption	First-in-human (case study)	194

## Abbreviations

AIDS: Acquired immunodeficiency syndrome
ARV: Antiretroviral
ART: Antiretroviral therapy
bNAbs: Broadly neutralizing antibodies
CAR T-cell: Chimeric antigen receptor T-cell
CRISPR: Clustered regularly interspaced short palindromic repeats
Cas9: CRISPR-associated protein 9
CCL5: C-C motif chemokine ligand 5
CCR5: C-C chemokine receptor type 5
CRS: Cytokine release syndrome
CTLA-4: Cytotoxic T-lymphocyte-associated protein 4
CXCL12: C-X-C chemokine ligand 12
CXCR4: C-X-C chemokine receptor type 4
CNS: Central nervous system
dCA: Didehydro-cortistatin A
HIV-1: Human immunodeficiency virus type 1
HSCs: Hematopoietic stem cells
HTLV-III: Human T-cell lymphotropic virus type III
iPSCs: Induced pluripotent stem cells
LASER-ART: Long-acting slow-effective release ART
LNs: Lymph nodes
LNPs: Lipid nanoparticles
LRAs: Latency-reversing agents
LTR: Long terminal repeat
MHC-I: Major histocompatibility complex class I
NIH: National institutes of health
PLWH: People living with HIV
PD-1: Programmed cell death protein 1
QVOA: Quantitative viral outgrowth assay
sgRNAs: Single-guide RNAs
SHIV: Simian-human immunodeficiency virus
SIV: Simian immunodeficiency virus
TALENs: Transcription activator-like effector nucleases
ULA ART: Ultra-long-acting antiretroviral therapy
Vpr: Viral protein R
ZFNs: Zinc-finger nucleases
